# MiRNA-based therapeutic intervention of cancer

**DOI:** 10.1186/s13045-015-0162-0

**Published:** 2015-06-11

**Authors:** Srivatsava Naidu, Peter Magee, Michela Garofalo

**Affiliations:** Transcriptional Networks in Lung Cancer Group, Cancer Research UK Manchester Institute, University of Manchester, Wilmslow Road, Manchester, M20 4BX UK

**Keywords:** Noncoding RNAs, Cancer therapy

## Abstract

MicroRNAs (miRNAs) are important modulators of eukaryotic gene expression. By targeting protein coding transcripts, miRNAs influence the cellular transcriptome and proteome, thus helping to determine cell fate. MiRNAs have emerged as crucial molecules in cancer research, in which recent studies have linked erratic expression of miRNAs to carcinogenesis and have provided solid evidence for their potential in cancer therapy. This review briefly summarises the recent knowledge on the involvement of miRNAs in tumourigenesis and reviews current studies on the therapeutic strategies and advances in the delivery of miRNAs.

## Introduction

MicroRNAs (miRNAs) are endogenous, small, noncoding RNAs that are highly conserved across various species of eukaryotes [1]. MiRNAs repress cellular translation and stability of a myriad of protein-coding transcripts by primarily targeting their 3′ untranslated regions (UTRs) in a sequence-specific manner [2, 3]. This selective silencing of gene expression by miRNAs has profound impact on human health and disease.

**Fig. 1:**
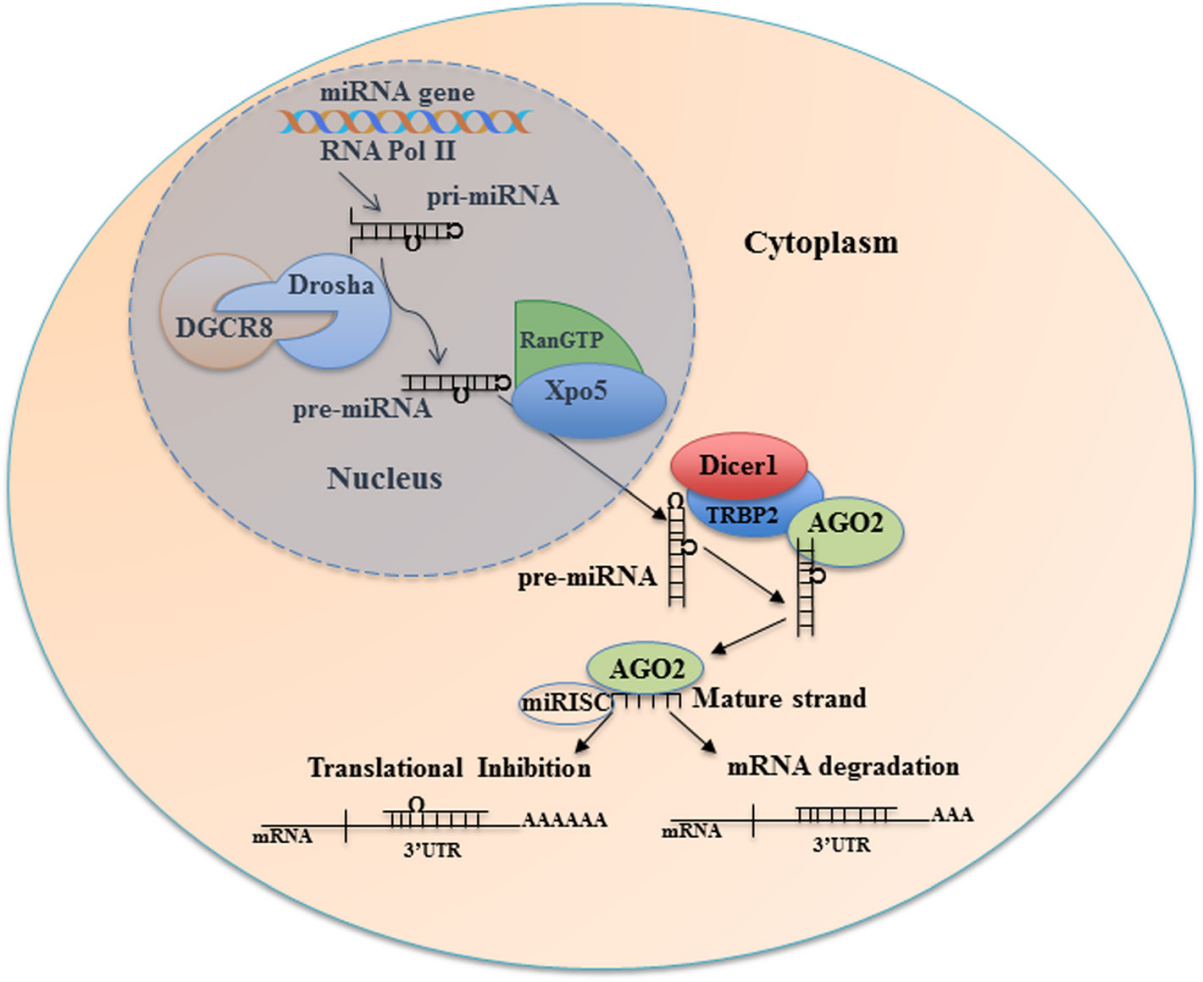
The majority of miRNA genes are transcribed by the RNA polymerase II (Pol II) as primary miRNAs (pri-miRNAs) which are processed to precursor miRNAs (pre-miRNAs) via the Drosha-DGR8 complex. Pre-mRNAs are exported to the cytoplasm by Exportin-5-Ran-GTP (Xpo-5-RanGTP) where Dicer1 cleaves the hairpin loop and Tar RNA-binding protein 2 (TARBP2) facilitates the RNA duplex loading onto Argonaute protein AGO2. AGO2 and the mature strand enter a protein effector complex formed by the RNA-induced silencing complex (miRISC). The miRNA guides the RISC to messenger RNA targets causing either mRNA cleavage (perfect complementarity) or translational repression (imperfect complementarity)

The latest release of miRBase [4] enlists at least 2588 miRNAs in humans. The canonical biogenesis of miRNAs is a tightly regulated process. Various epigenetic, transcriptional and processing mechanisms fine-tune the spatial and temporal expression of miRNAs [5]. MiRNA genes are predominantly transcribed by RNA polymerase II as primary miRNAs (pri-miRNAs) which are processed to precursor miRNAs (pre-miRNAs) in the nucleus by a microprocessor complex (composed of Drosha and DGCR8 (DiGeorge syndrome critical region 8)) [6]. Subsequently, pre-miRNAs are exported to the cytoplasm by Exportin-5-Ran-GTP complex [7], where Dicer1 cleaves the hairpin loop of pre-miRNA [8, 9] and TARBP2 (TAR RNA-binding protein 2) facilitates RNA duplex loading onto Argonaute protein AGO2 [10]. The antisense strand (mature) is retained by AGO2 and the sense strand is degraded, thus configuring a silencing complex [11] (Fig. 1). Non-canonical miRNA biogenesis has also been reported, and these mechanisms are reviewed elsewhere [12].

MiRNAs silence gene expression via multiple mechanisms [13]. The current model suggests that miRISC (miRNA-induced silencing complex) binds to the complementary “seed” region within the 3′ UTR of target mRNAs (messenger RNAs) and influences their degradation and/or the level of translation [3]. The thermodynamic stability of the miRNA-mRNA interaction is critical for effective repression of a potential target; however, other factors, including RNA secondary structure and spatial constraints posed by the same or other miRNA binding sites within the 3′ UTR, may influence the silencing outcome of the target [14] (Fig. 1). Exponentially growing evidence confers the unique potential of miRNAs to modulate diverse biological processes, including cell growth and proliferation, cell cycle control, differentiation, apoptosis and tissue development [15, 16]. Therefore, it is highly plausible that any aberration or deregulation in miRNA expression can be detrimental to the cell. Further, numerous studies have intimately linked erratic miRNA expression to the aetiology of cancer. Here, we briefly summarise the recent evidence for the involvement of miRNAs and miRNA-based therapeutic strategies in various cancers. Also, we highlight advances on the therapeutic delivery of miRNAs for the treatment of cancer.

### MiRNAs in cancer: a friend and foe

The role of miRNAs in cancer was first reported by Calin et al. in chronic lymphocytic leukaemia [17]. Subsequently, a plethora of studies strongly correlated the deregulated expression of miRNAs in the hallmarks of cancer [18]. Diverse cellular mechanisms contribute to miRNA deregulation in cancer, and genetic changes [19], aberrant DNA methylation [20] and histone acetylation [21] have been attributed to miRNA deregulation. Importantly, cancer-related transcription factors such as myc [22] and p53 [23] have been shown to influence miRNA expression. Additional mechanisms including alternative splicing, polyadenylation and mutations in miRNA processing machinery may also hamper miRNA maturation [24]. Aberrant loss or gain of miRNAs contributes to initiation, progression, metastasis and drug resistance of a wide spectrum of cancers. Depending on the genes and/or pathways they affect, miRNAs can act as tumour suppressors or oncogenes in a tissue-specific manner. For example, let-7 family miRNAs are known to be tumour suppressors. Downregulation of let-7 expression has been reported in head, neck, lung, breast, ovarian and prostate cancers [25]. Let-7 negatively regulates oncogenes such as KRAS, c-MYC, CDK6, HOXA9, TGFBR1, BCL-XL and MAP4K3, thereby promoting anti-oncogenic pathways [26]. Similarly, miR-34 family [27], miR-223 [28], miR-143/145 cluster [29, 30] and miR-204 [31] are commonly downregulated in various cancers; interestingly, reconstitution of respective miRNAs in these studies significantly reduced tumour growth. Interestingly, miR-214 is oncogenic in osteosarcoma [32] and nasopharyngeal cancer [33], whereas it appears to be a tumour suppressor in glioma [34] and colorectal cancer [35]. Similarly, miR-125b displays an oncogenic phenotype in colon and haematopoietic cancers, whereas it acts as a tumour suppressor in breast cancer and hepatocellular carcinoma [36]. Additional miRNAs, namely miR-17/92 cluster, miR-21, miR-155, miR-221, miR-222 and miR-9 are upregulated in various cancers [37]. Elevated miR-17/92 levels caused oncogenic activation of PI3K and NF-kB signalling in lymphomas [38]. MiR-21 overexpression has a causal role in tumourigenesis of pre-B cell lymphomas [39]. MiR-155 inhibition restored expression of tumour suppressor TP53INP1 and inhibited tumour development in breast cancer [40]. The intimate role of miRNAs in tumour metastasis, drug resistance and cancer stemness has been discussed elsewhere [41]. A more recent update on the role of miRNAs in various cancers has been tabulated (Table 1). Taken together, dysregulated miRNA expression appears to influence various hallmarks of cancer. A comprehensive understanding of miRNA biology in carcinogenesis can possibly pave novel routes for anti-cancer therapy.

**Table 1:** A list of miRNAs involved in various cancer types. Corresponding functional role (phenotype) and validated targets (targets) are shown in separate columns

Cancer	miRNA	Phenotype	Targets	Reference
Lung cancer	miR-132/212	TS	CyclinD1	[72]
(NSCLC)	miR-124	TS	SOX8	[73]
	miR-126	TS	VEGF-PI3K-Akt-MRP1	[74]
	miR-181	TS	Bcl2	[75]
	miR-34a	TS	TGFβR2	[76]
	miR-145	TS	Oct-4	[77]
	miR-21	OG	PDCD4	[78]
	miR-137	PM	SLC22A18	[79]
Gastro-intestinal cancers				
Gastric cancer	miR-335	TS	RASA1	[80]
	miR-374b-5p	OG	RECK	[81]
	miR-490-3p	OG	SMARCD1	[82]
	miR-199a-3p	OG	ZHX1	[83]
Colorectal cancer	miR-185	TS-PM	STIM1	[84]
	miR-92a	OG-PM	PTEN	[85]
	miR-7	TS-PM	EGFR	[86]
Hepatocellular carcinoma	miR-9	PM		[87]
	miR-150-5p	TS	MMP14	[88]
	miR-21	OG-PM	AP1	[89]
	miR-122	TS	Hnf4α-GALNT10-EGFR	[90]
	miR-486-5p	TS	PIK3R1	[91]
Esophageal cancer	miR-101, miR-127	TS	MALAT1	[92]
	miR-126	TS	DNMT1/ADAM9-EGFR	[93]
	miR-27a	TS	K-Ras	[94]
Haematological cancers				
Lymphoma	miR-155-3p,	TS	LT-β	[95]
	miR-224	TS-PM	CD59	[96]
	miR-17-92	OG	Sin3b, Hbp1, Suv420h1, Btg1, Bim	[97]
Leukaemia	miR-486-5p	OG	AKT-FOXO1	[98]
	miR-22	OG	PTEN	[99]
	miR-638	TS	CDK2	[100]
Reproductive cancers				
Cervical cancer	miR-126	TS-PM	PTEN	[101]
	miR-21, Let-7a	OG/TS	STAT3	[102]
	miR-375	DR	E-cadherin	[103]
Prostate cancer	miR-3195, miR-374b	TS	HIF-1α, HIF-2α and VEGF	[104]
	miR-218	TS	TPD52	[105]
	miR-449b	PM		[106]
Breast cancer	miR-873	TS-DR	ERα–CDK3	[107]
	miR-18b, miR-103, miR-107 and miR-652	PM		[108]
	miR-7	TS-DR	EGFR, Src kinase	[109]
Glioblastoma	miR-125a-5p	TS	TAZ	[110]
	miR-155	OG-DR	MAPK13 and MAPK14	[111]
	miR-449a	TS	MAZ	[112]
	miR-148a	TS	Oct4, Sox-2	[113]

Abbreviations: *NSCLC* non-small cell lung cancer; *TS* tumour suppressor; *OG* oncogenic; *PM* prognostic marker; *DR* drug resistance

### MiRNAs in cancer therapy: the potential

MiRNAs are ubiquitously deregulated, as a cause or consequence, in virtually all cancers. By modulating multiple targets or entire pathways, and by having unique expression profiles and higher stability in biological samples, miRNAs have quickly gained diagnostic and therapeutic value. Modulating miRNA expression for cancer therapy is currently under investigation; in general, the therapeutic modulation of miRNAs is achieved by inhibiting oncogenic miRNAs, or by reconstituting tumour suppressor miRNAs. In recent years, the therapeutic potential of miRNAs in cancer has been demonstrated in several published studies (Table 1). For this review, we only focus on the most recent in vivo and preclinical studies which employed, either alone or in combination with conventional drugs, miRNAs as active agents against various cancers.

Xue et al. demonstrated that targeted delivery of miR-34a and K-ras siRNA into a murine lung cancer model resulted in significant tumour regression. Reconstitution of miR-34a reduced the mRNA levels of oncogenes such as Ccnd1, Sirt1, Cdk6 and Ccne2. Furthermore, modulating RNA delivery combined with cisplatin prolonged mice survival in this model [42]. In an independent study, combined reconstitution of miR-34 and let-7 reduced the expression of the tumour promoters Lin28b, c-Met and Myc and, as a consequence, tumour growth was drastically decreased in a murine model of non-small cell lung cancer (NSCLC) [43]. Another study reported that restoring miR-200c in a xenograft model enhanced sensitivity of lung tumours to radiation by targeting the expression of DNA repair protein RAD51 and oxidative stress response genes peroxiredoxin-2, Nrf2 and SESN1 [44]. Together, these in vivo models strongly support miRNAs as novel therapeutic options, either alone or in combination, for treating deadly lung cancers which are often presented with few treatment options.

Interestingly, two independent studies have demonstrated that targeted delivery of miR-520e [45] and miR-375 [46] dramatically reduced liver cancer cell growth in in vivo xenograft models. Independently, ectopic expression of miR-217 significantly reduced tumour growth in a pancreatic ductal adenocarcinoma (PDAC) xenograft model [47]. Conversely, knockdown of oncogenic miR-21 expression combined with gemcitabine has been shown to be effective in controlling pancreatic ductal adenocarcinoma in a mouse model [48]. In addition, forced expression of miR-25 into a colon cancer mice model markedly reduced tumour burden [49]. Intriguingly, miR-182 expression promoted metastasis by modulating the expression of endothelial-mesenchymal transition (EMT) components in colorectal cancer; however, inhibition of miR-182 reversed this effect [50]. Taken together, these studies warrant the therapeutic benefits of miRNAs in the treatment of various gastro-intestinal cancers.

Liu et al. have shown that targeted downregulation of miR-106b-5p and its targets retinoblastoma-like 1 and 2 (RBL1, 2) and caspase-8 inhibited glioma formation in a xenograft mice model [51]. Conversely, ectopic expression of miR-1 in glioblastoma-derived extracellular vesicles halted tumour growth, neovascularization and invasiveness [52]. Also, miR-142-3p overexpression modulated cytokine signalling in disease-associated infiltrating macrophages, resulting in glioma tumour growth inhibition [53]. These reports highlight the beneficial role of miRNAs in otherwise untreatable glioma.

Specific knockdown of miR-20b in a breast cancer nude mice model has shown to suppress tumour growth in vivo [54]. Intriguingly, a recent study demonstrated that in vivo delivery of miR-31 resulted in increased sensitivity for paclitaxel in an ovarian cancer model [55]. In the light of above studies, modulating miRNA expression appears to be a promising strategy for cancer therapy; however, caveats such as off-target effects, functional redundancy and dual nature (tumour promoter and tumour suppressor) should be considered carefully before utilising miRNAs for anti-cancer therapies.

### MiRNAs in cancer therapy: the challenge

Successful translation of miRNAs into cancer therapeutics heavily depends on the specific, efficient and safe delivery of miRNA modulators to the tumour sites. Various biological barriers including in vivo nuclease degradation, fibrous nature of tumours, insufficiency of miRNA processing machinery and miRNA-induced immune response drastically hinder the bioavailability of ectopic miRNAs. Multiple approaches are in rapid development to circumvent these delivery hurdles. Chemical modifications on 2′-OH ribose [56] or phosphate backbone (locked nucleic acids, or LNAs) [57] made synthetic miRNAs less vulnerable to nuclease degradation, thus increasing in vivo stability and affinity to the target sequence. Various strategies have been developed for efficient delivery of miRNA modulators. Lentiviral, adenoviral and adeno-associated viral (AAVs) vectors expressing miRNA antagonists or mimics have proven to be effective delivery systems in various cancer models [58]. Importantly, cell-specific moieties can be engineered onto the viral capsid to enhance specificity uptake by cancer cells [59]. Non-integrating AAVs were successfully used as vehicles for miRNA replacement therapy in liver cancer [60]. Recently, exosomes and vesicles released by virus-infected cells have been shown to encapsulate and deliver miRNAs into the target cells, indicating that viral-derived exosomes can be exploited for miRNA delivery [61]. Another approach of vectors expressing tandem repeats of miRNA antisense sequences, termed miRNA sponges, can de-repress miRNA targets and have shown to be effective in modulating miRNA expression in vitro and in vivo [62], [63]. This approach offers a unique opportunity to target a family of miRNAs sharing the same seed sequence. A recent study engineered a vector expressing miRNA sponges and claims that this vector could circumvent some of the technical problems associated with this method [64]. Technological and engineering advancements have generated a variety of nanomaterials for miRNA delivery. Specifically, nanosized gold, carbon and silica particles have been used as carriers for miRNAs. Recently, systemic delivery of miR-34a-silica nanoparticles coated with tumour-specific antibody led to significant tumour growth inhibition and enhanced apoptosis in neuroblastoma [65]. Similarly, owing to their biodegradable property and high electrostatic affinity towards cellular membranes, cationic polymers have become an attractive option for miRNA transport. Poly-lacticco-glycolic acid (PLGA), polyethylenimine (PEI) and poly-dimethylaminoethyl methacrylate (PDMAEMA) are common cationic polymers used in nucleic acid delivery for cancer therapy. For example, systemic delivery of PLGA-based miR-21 and miR-10b antagonists in a breast cancer model caused dramatic effects on tumour regression [66]. Encouragingly, PEI-based miR-145 nanoparticles combined with radiation and chemotherapy significantly eliminated metastatic tumour nodules in a lung adenocarcinoma mice model [67]. Furthermore, Trang et al. successfully delivered lipid nanoparticles harbouring miR-34a or let-7 mimics which reduced tumour size in a K-ras-activated NSCLC mouse model [68]. Recently, advances in nanotechnology have led to an integrated diagnostic and therapeutic platform called “nanotheranostics” [69]. This novel system offers a unique possibility to load both diagnostic and therapeutic agents onto a single nanoparticle, thus facilitating to monitor the distribution, release and efficacy of drug in real time. Given the immense potential of miRNAs as biomarkers and therapeutic molecules, miRNA-based nanotheranostics could open novel avenues for personalised medicine in cancer therapy. Although current miRNA delivery methods (Table 2) have been promising in various experimental models, each of these systems suffers from various limitations. Despite the high transfection efficacy, viral-based delivery methods may elicit immune/inflammatory response, and may also suffer from the risk of integrating into the host genome, leading to oncogenic insertional mutagenesis. Nevertheless, advanced genetic engineering methods may lead to improved safety profiles for further exploiting these methods for therapeutic miRNA delivery. On the other hand, challenges such as cytotoxicity, non-specific tissue distribution and high cost of production remains to be addressed for the nanoparticle-based delivery platforms. Therefore, a reliable, safe and cost-effective miRNA delivery strategy would largely be beneficial for exploiting miRNAs for clinical purposes.

**Table 2:** A summary of various miRNA delivery methods and their potential modulatory effects

Delivery method	Modulatory effect
Viral based	Replacement/inhibition
Lentiviral	
Adenoviral	
Adeno-associated viral	
Viral derived exosomes	
Nanoparticle based	Replacement/inhibition
Inorganic	
Gold particles	
Carbon particles	
Silica particles	
Organic polymers	
Poly-lacticco-glycolic acid	
Polyethylenimine	
Poly-dimethylaminoethyl- methacrylate	
Lipid nanoparticles	
Sponges	Inhibition

### Future perspectives

Given the heterogeneous nature of the disease and the limitations facing conventional therapies, cancer therapy needs to be multidimensional. Research so far has undoubtedly established the potential of miRNAs in diagnosis, therapy and prognosis of cancer. Successful in vivo and preclinical studies have appreciated the therapeutic potential of miRNAs for cancer treatment. For instance, restoring the expression of miRNAs such as Let 7, miR-16 and miR-31 has shown to have anti-cancer effects in various preclinical models [70]. Thus far, studies have proposed several miRNAs as potential candidates for cancer therapy [71]. In particular, miR-34, a key tumour suppressor miRNA, appears to be promising. Ectopic expression of miR-34 has shown to have therapeutic benefits for a variety of cancers [27]. Moreover, a liposome-based mimic of miR-34 (MRX34) developed by Mirna Therapeutics recently progressed into phase I clinical trials (identifier: NCT01829971). The outcome of this trial (due in December 2015) may hopefully provide more insights into this novel paradigm. Although technical challenges remain to be addressed, miRNA therapeutics appear to hold huge promise for cancer treatment, at least for those cancers where other treatment options have plateaued. Comprehensive understanding of the role of miRNAs in complex regulatory networks involved in cancer may help with designing combinatorial therapeutic strategies. Further developments in miRNA delivery technologies will hopefully translate miRNA-based cancer therapeutics into a clinical reality.
